# Polysplenia Syndrome With Splenic and Skeletal Muscle Metastases From Thyroid Carcinoma Evaluated by FDG PET/CT: Case Report and Literature Review: A CARE-Compliant Article: Erratum

**DOI:** 10.1097/01.md.0000494756.80250.4a

**Published:** 2016-08-26

In the article “Polysplenia Syndrome With Splenic and Skeletal Muscle Metastases From Thyroid Carcinoma Evaluated by FDG PET/CT: Case Report and Literature Review: A CARE-Compliant Article”,^1^ which appeared in Volume 95, Issue 4 of *Medicine*, the date of manuscript acceptance was incorrectly given as December 21, 2016. The correct date was December 21, 2015. The article has since been corrected online.
